# Do quantitative and qualitative shear wave elastography have a role in evaluating musculoskeletal soft tissue masses?

**DOI:** 10.1007/s00330-016-4427-y

**Published:** 2016-06-08

**Authors:** B. Pass, M. Jafari, E. Rowbotham, E. M. A. Hensor, H. Gupta, P. Robinson

**Affiliations:** 1 Musculoskeletal Centre X-Ray Department, Leeds Teaching Hospitals Trust, Chapel Allerton Hospital, Leeds, UK; 2 Chapel Allerton Hospital, University of Leeds and NIHR Leeds Musculoskeletal Biomedical Research Unit, Leeds, UK

**Keywords:** Ultrasound, Sarcoma, Imaging, Elasticity imaging techniques, Soft tissue neoplasms

## Abstract

**Objectives:**

To determine if quantitative and qualitative shear wave elastography have roles in evaluating musculoskeletal masses.

**Methods:**

105 consecutive patients, prospectively referred for biopsy within a specialist sarcoma centre, underwent B-mode, quantitative (m/s) and qualitative (colour map) shear wave elastography. Reference was histology from subsequent biopsy or excision where possible. Statistical modelling was performed to test elastography data and/or B-mode imaging in predicting malignancy.

**Results:**

Of 105 masses, 39 were malignant and 6 had no histology but benign characteristics at 12 months. Radiologist agreement for B-mode and elastography was moderate to excellent Kw 0.52-0.64; PABAKw 0.85-0.90). B-Mode imaging had 78.8% specificity, 76.9% sensitivity for malignancy. Quantitatively, adjusting for age, B-mode and lesion volume there was no statistically significant association between longitudinal velocity and malignancy (OR [95% CI] 0.40[0.10, 1.60], p=0.193), but some evidence that higher transverse velocity was associated with decreased odds of malignancy (0.28[0.06, 1.28], p=0.101). Qualitatively malignant masses tended to be towards the blue spectrum (lower velocities); 39.5% (17/43) of predominantly blue masses were malignant, compared to 14.3% (1/7) of red lesions.

**Conclusions:**

Quantitatively and qualitatively there is no statistically significant association between shear wave velocity and malignancy. There is no clear additional role to B-mode imaging currently.

***Key Points*:**

*• Correlation between shear wave velocity and soft tissue malignancy was statistically insignificant*

*• B-mode ultrasound is 76.9 % sensitive and 78.8 % specific*

*• Statistical models show elastography does not significantly add to lesion assessment*

## Introduction

Soft tissue sarcomas constitute less than 1 % of all malignancies. [1] Soft tissue masses have a benign to malignant ratio of over 100:1 [2, 3], meaning that a large number of lesions that are ultimately benign will undergo imaging investigation and biopsy.

Elastography uses external compression to determine tissue strain and lesion stiffness [4]. The more recent development of acoustic radiation force impulse (ARFI) imaging does not require operator external compression, and thus should give less variability. Soft tissues are transiently deformed using acoustic radiation and the measured dynamic displacement is used to estimate the tissue's mechanical properties. [5] It has been established that benign breast and prostate masses tend to be soft and malignant masses tend to be stiff. [6] In breast imaging, elastography has been shown to improve specificity [7] and potentially further characterise B-mode-detected breast lesions [8] with highly reproducible results [9].

In musculoskeletal imaging, ultrasound (US) is well established as a diagnostic tool [10, 11] for initially assessing clinically suspicious soft tissue masses [12]. The role of shear wave elastography in musculoskeletal imaging has been largely limited to the evaluation of tendon disorders [13–15].

There is only limited published data on the role of sonoelastography in the evaluation of soft tissue masses [16, 17]. The aim of this study was to determine whether quantitative and qualitative shear wave elastography when assessed along with B-mode US imaging could have a role in the evaluation of musculoskeletal soft tissue masses.

## Materials and methods

### Patient population

The institutional ethics committee approved the study protocol and all patients underwent informed consent. Consecutive patients were prospectively referred from the specialist sarcoma service over a nine-month period for soft tissue biopsy of an extremity soft tissue mass with no clinical exclusion criteria.

### B-Mode US imaging

All B-mode imaging was performed by one of three experienced consultant musculoskeletal radiologists (15, six, and four years experience) using US (Siemens Acuson S3000, Erlangen, Germany) and a linear array transducer (9-4 MHz).

B-mode US findings (echogenicity compared to muscle, homogenous vs heterogeneous), lesion size (3 dimensions, cm), vascularity (power Doppler recorded as absent, present linear or present disorganised) and lesion position (subcutaneous, deep intramuscular or deep intermuscular) were documented for all patients.

At a subsequent sitting over 1 month following the initial study, the anonymised B-mode images were reviewed independently by the three radiologists blinded to the other forms of imaging, elastography, and histology. The B-mode images were subjectively classified, using previously described B-mode criteria [12] into a four-point system assessing the likelihood of the lesion being benign or malignant; 1 = definite benign, 2 = indeterminate benign, 3 = indeterminate malignant, 4 = definite malignant. Discrepancies between scores were then resolved by consensus.

### SW Elastographic imaging

**Fig. 1:**
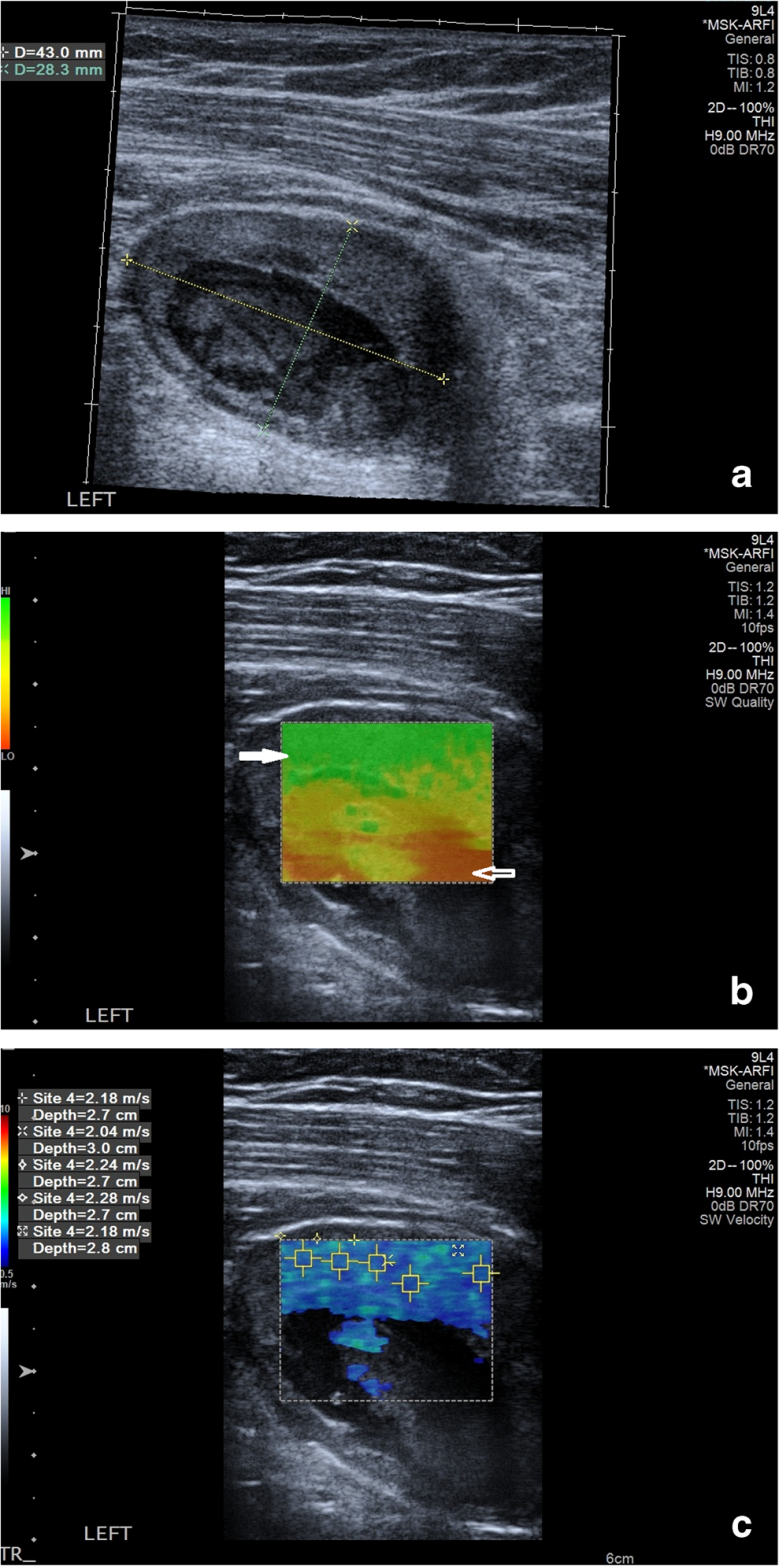
Glomus tumour in a 62-year-old patient. (**a**) Transverse B-mode image shows an oval mass with solid vascular periphery and necrotic/cystic centre. (**b**) Corresponding shear wave velocity quality map with the solid area green (good quality reading) (solid arrow) and cystic/necrotic area red (poor quality reading)(hollow arrow). (**c**) Corresponding shear wave qualitative map, which is predominantly blue/cyan with five quantitative readings

Prior to biopsy all patients underwent acoustic radiation force impulse (ARFI) imaging (Virtual Touch Quantification; Siemens) where shear waves are generated after the application of the focused push pulse while holding the probe lightly on the skin surface and the adjacent muscles are relaxed [18, 19]. The user defined a region of interest (ROI) and the software synthesised information from 256 sequential acquisition beam lines to generate qualitative and quantitative shear wave velocity maps with the velocity range kept constant at 0 - 10 metres per second (m/s) [18]. Initially, a quality map was produced displaying the quality range of the readings with green representing good quality and red meaning bad quality (Fig. 1). Shear wave velocity colour maps in both longitudinal and transverse planes were obtained and five quantitative readings taken using 2 × 2 mm ROIs in the most homogenously solid (and vascular when present) area of the mass, which demonstrated good quality readings on the accompanying quality map. The mean of the five velocity readings was used for further statistical analysis. In 28 patients, colour maps and velocity readings were repeated independently by a second operator blinded to the initial readings to allow inter-observer assessment.

The anonymised colour shear wave velocity maps were evaluated with colour percentage image processing software (Image J analysis in Java, National Institute of Health) documenting five categories: red (high velocity), orange/yellow, yellow/green, cyan and blue (lowest velocities) relating to the dominant distribution of shear wave elastography readings. Any areas within the map returning no colour (e.g. cystic or necrotic areas) were not included in the software analysis.

Masses subsequently underwent percutaneous biopsy, excision, or both, and histological diagnosis (and where appropriate molecular and cytogenetic analysis) was taken as the reference standard.

### Statistical analysis

Based on pilot study data, we anticipated that 30 % of lesions would be malignant. Assuming that we wished to control for up to four independent variables in a logistic regression model, to satisfy rules of thumb requiring 10 events per variable, the study designed required n = 135 patients to be recruited [20].

For lesions that were located both subcutaneously and extending into deep tissues, if any part of the lesion extended into the muscle, it was coded intramuscular (n = 3), otherwise it was coded intermuscular (n = 1). Mass volume was calculated assuming an ellipsoid shape as π/6 x craniocaudal (hereafter longitudinal) x anteroposterior x transverse. Prior to statistical analysis, right-skewed continuous variables (shearwave velocity and lesion volume) were natural log-transformed. Shearwave colour map data (proportions of pixels that were red, orange, yellow, cyan, or blue) were used to determine the dominant colour of the lesion. Intra-reader reliability of velocity measurements was assessed using intraclass correlation coefficients (ICCs); agreement over B-mode classification and dominant lesion colour were assessed using quadratic-weighted Kappa (Kw), prevalence-adjusted bias-adjusted quadratic-weighted Kappa (PABAKw), and proportions of positive agreement. Spearman’s rho coefficients were calculated to assess associations among map colour proportions and mean shearwave velocities; Pearson’s r coefficients were calculated for associations among ln-transformed shear wave velocities and volume. Analysis of variance (ANOVA) was used to test the extent to which the dominant map colour was associated with shearwave velocity. Kruskal-Wallis test was used to compare the ratio of longitudinal:transverse velocity between lesions located within muscles, between muscles, or solely in subcutaneous tissue. Linear regression was used to model associations between B-mode findings and shear wave velocity. Binary logistic regression was used to determine the unadjusted and adjusted odds of malignancy based on the B-mode findings and shear wave velocities, adjusted for demographic variables. Appropriate checks were made that test assumptions were met. The four B-mode consensus categories were collapsed into ‘definitely or indeterminately benign’ and ‘definitely or indeterminately malignant’ to calculate sensitivity and specificity. Pseudo R-squared and Akaike’s Information Criterion (AIC) were calculated to compare the predictive strengths of different models. Bootstrapping using 2000 replicates was used to obtain stable standard error estimates for regression coefficients.

All analyses were conducted in Stata 13.1 (StataCorp. 2013. Stata Statistical Software: Release 13. College Station,TX).

## Results

The original recruitment target was n = 135; however, due to limited equipment availability, n = 114 patients were recruited. Of these 114 patients, nine did not have shear wave measurements available and were excluded from analysis. Of the remaining 105 patients, in six cases no biopsy was performed because of patient choice (n = 5) or technically difficult biopsy (n = 1). All had benign imaging appearances and the lesions were assumed to be benign for the purposes of analysis, since at follow-up imaging (12 months) all lesions had remained unchanged (n = 5) or resolved (n = 1, haematoma). Demographic characteristics and US findings for the 105 patients are summarised in Table 1. Although this fell short of the intended sample size of 135, with 39 malignant lesions the event-per-variable ratio was still approximately 10:1 in our multivariable logistic regression models of malignancy, which contained up to four variables. Biopsy confirmed malignant lesions in 39 (37.1 %).

**Table 1:** Demographic characteristics and US findings in patients with shear wave velocity measurements available (n = 105)

Continuous variable	All patients (n = 105)
Age, years	
Mean (SD)	52.3 (18.3)
Range	20 to 88
Sex	
Male % (n)	54.3 % (57)
Longitudinal SW velocity, m/s	
Geometric mean	2.80
Median (IQR)	2.54 (1.94, 4.12)
Range	0.82 to 9.65
Longitudinal SW colour	
Red	7.7 % (7/91)
Orange	- (0/91)
Yellow	30.8 % (28/91)
Cyan	14.3 % (13/91)
Blue	47.3 % (43/91)
Transverse SW velocity, m/s	
Geometric mean	2.79
Median (IQR)	2.42 (1.94, 4.30)
Range	1.01 to 9.78
Transverse SW colour	
Red	10.8 % (10/93)
Orange	- (0/93)
Yellow	29.0 % (27/93)
Cyan	7.5 % (7/93)
Blue	52.7 % (49/93)
Mass volume, cm^3^	
Geometric mean	29.2
Median (IQR)	24.90 (10.08, 92.36)
Range	0.20 to 3247.43
Lesion position	
Subcutaneous only % (n)	28.6 % (30)
Deep % (n)	71.4 % (75)
Inter-muscular % (n)	35.2 % (37)
Intra-muscular % (n)	36.2 % (38)
Mass quality	
Heterogeneous % (n)	65.7 % (69)
Necrotic % (n)	28.6 % (30)
Echogenicity	
Hyperechogenic % (n)	8.6 % (9)
Power Doppler signal	
Present % (n)	61.0 % (64)
Linear % (n)	24.8 % (26)
Disorganised % (n)	35.2 % (37)
Mixed % (n)	1.0 % (1)

### Reliability of US classification

Radiologist agreement for US diagnosis was moderate, but was excellent when the effects of prevalence and bias were taken into account (Kw 0.52-0.64; PABAKw 0.85-0.90). Compared to the eventual consensus classification, for each individual reader the agreement was substantial (Kw = 0.74-0.82) and each reader over- and under-estimated the consensus classification in no more than 16 % of cases in each direction.

### Associations between B-mode findings and consensus classification

A binary logistic regression model of the individual US findings, in which the collapsed consensus classification (benign vs malignant) was the outcome, showed that the odds of a lesion being classified as indeterminate malignant or malignant were not substantively associated with lesion heterogeneity or lesion position (Table 2). The odds were substantively higher for necrotic masses, although not statistically significant. Hyperechogenic lesions were less likely to be considered malignant, whilst the odds were slightly raised for linear and raised considerably for lesions with a disorganised power Doppler signal.

**Table 2:** Associations between US findings and collapsed consensus classification of lesions as benign (benign, indeterminately benign) or malignant (indeterminately malignant, malignant)

US characteristic	Collapsed consensus classification		Odds ratio^1^ (95 % CI)	*P* value
	Benign	Malignant		
Mass texture				
Homogeneous	72.2 % (26)	27.8 % (10)	Reference	
Heterogeneous	50.7 % (35)	49.3 % (34)	1.20 (0.33, 4.36)	0.777
Necrosis				
Absent	65.3 % (49)	34.7 % (26)	Reference	
Present	40.0 % (12)	60.0 % (18)	1.63 (0.41, 6.42)	0.488
Echogenicity				
Hypo or mixed	55.2 % (53)	44.8 % (43)	Reference	
Hyper	88.9 % (8)	11.1 % (1)	0.20 (0.05, 0.82)	0.025
Power Doppler				
Absent	80.5 % (33)	19.5 % (8)	Reference	
Linear	59.3 % (16)	40.7 % (11)	2.19 (0.53, 9.09)	0.282
Disorganised	32.4 % (12)	67.6 % (25)	7.79 (1.90, 31.94)	0.004
Position				
Subcutaneous	56.7 % (17)	43.3 % (13)	Reference	
Intermuscular	51.4 % (19)	48.7 % (18)	1.11 (0.27, 4.49)	0.883
Intramuscular	65.8 % (25)	34.2 % (13)	0.57 (0.12, 2.82)	0.492

^1^From multivariable logistic regression model including all US characteristics

### Sensitivity and specificity of consensus 2D scoring for malignancy

**Fig. 2:**
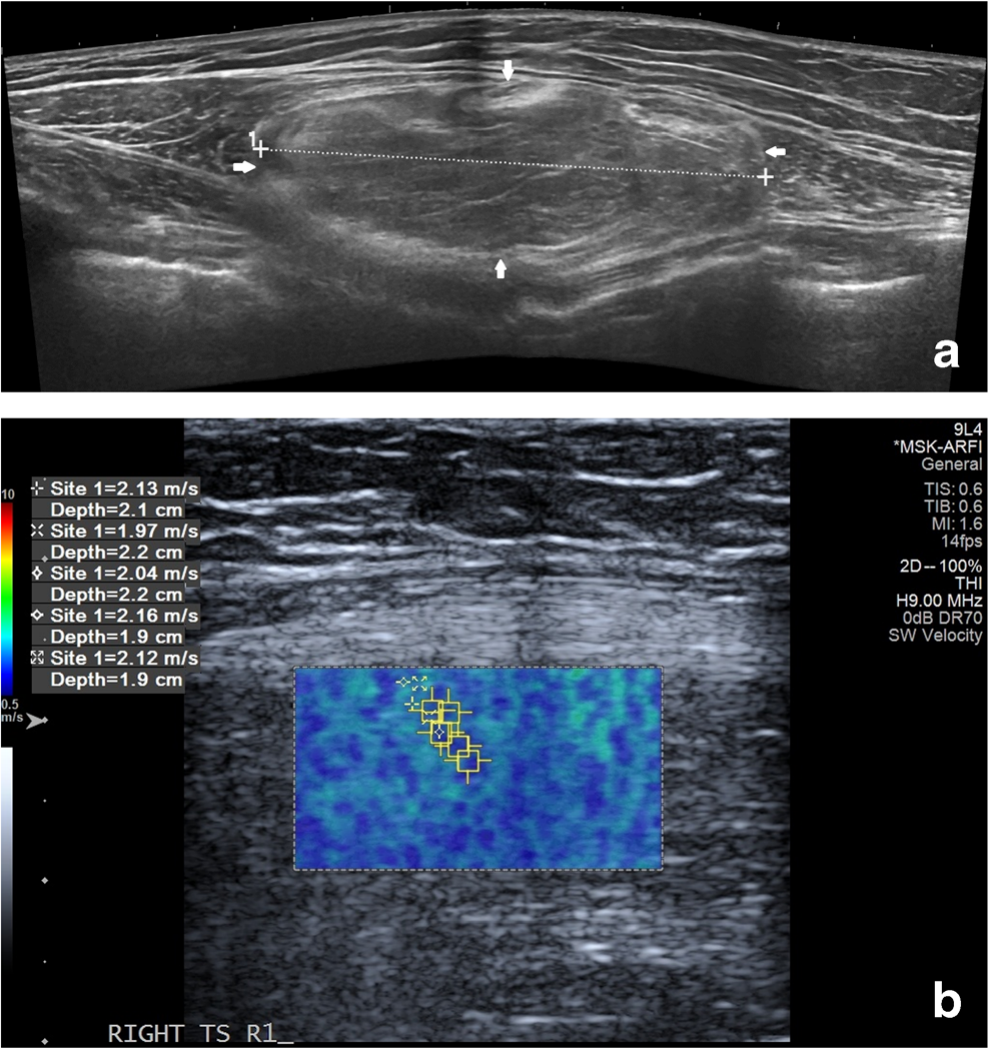
Liposarcoma grade 1 in a 79-year-old patient**.** (**a**) Longitudinal B-mode panoramic image showing well-demarcated subfascial intramuscular lipomatous lesion (solid arrows). (**b**) Corresponding shear wave qualitative map, which is predominantly blue/cyan with five quantitative readings performed in transverse plane on small area of the superficial aspect of the lesion

After collapsing the four consensus categories, sensitivity (Wilson 95 % CI) was 76.9 % (61.7 %, 87.4 %) and specificity was 78.8 % (67.5 %, 86.9 %) (Table 3). However, 9/39 malignant masses (23.1 %) were classified as definitely or indeterminately benign on the 2D consensus score (n = 8 low grade sarcomas, n = 1 high grade pleomorphic sarcoma) (Fig. 2).

**Table 3:** Proportions of masses found to be benign or malignant according to the results of 2-D consensus scoring

2-D scoring consensus	Benign (True negative) n = 66	Malignant (True positive) n = 39
Definitely benign	7.6 % (5)	2.6 % (1)
Indeterminate (benign)	71.2 % (47)	20.5 % (8)
Indeterminate (malignant)	21.2 % (14)	53.9 % (21)
Malignant	- (0)	23.1 % (9)
Benign (2-D score 1 or 2)	78.8 % (52)	23.1 % (9)
Malignant (2-D score 3 or 4)	21.2 % (14)	76.9 % (30)

### Reliability of shear wave velocity measurements

The shear wave readings repeated in 28 patients suggested they were highly reproducible (longitudinal ICC [95 % CI] 0.89 [0.79, 0.95]; transverse 0.98 [0.96, 0.99]). The ICCs for average values from two readers were 0.94 (0.88, 0.97) and 0.99 (0.98, 1.00), respectively.

### Reliability of shear wave colour map measurements

Repeated colour map measurements were available for n = 26 patients (missing in two cases due to a data fault). Agreement for predominant colour map proportions was substantial in both planes (Kw [95 % CI] longitudinal 0.84 [0.56, 0.96]; transverse 0.81 [0.58, 0.94]).

### Patterns in the data with respect to shear wave velocity

**Fig. 3:**
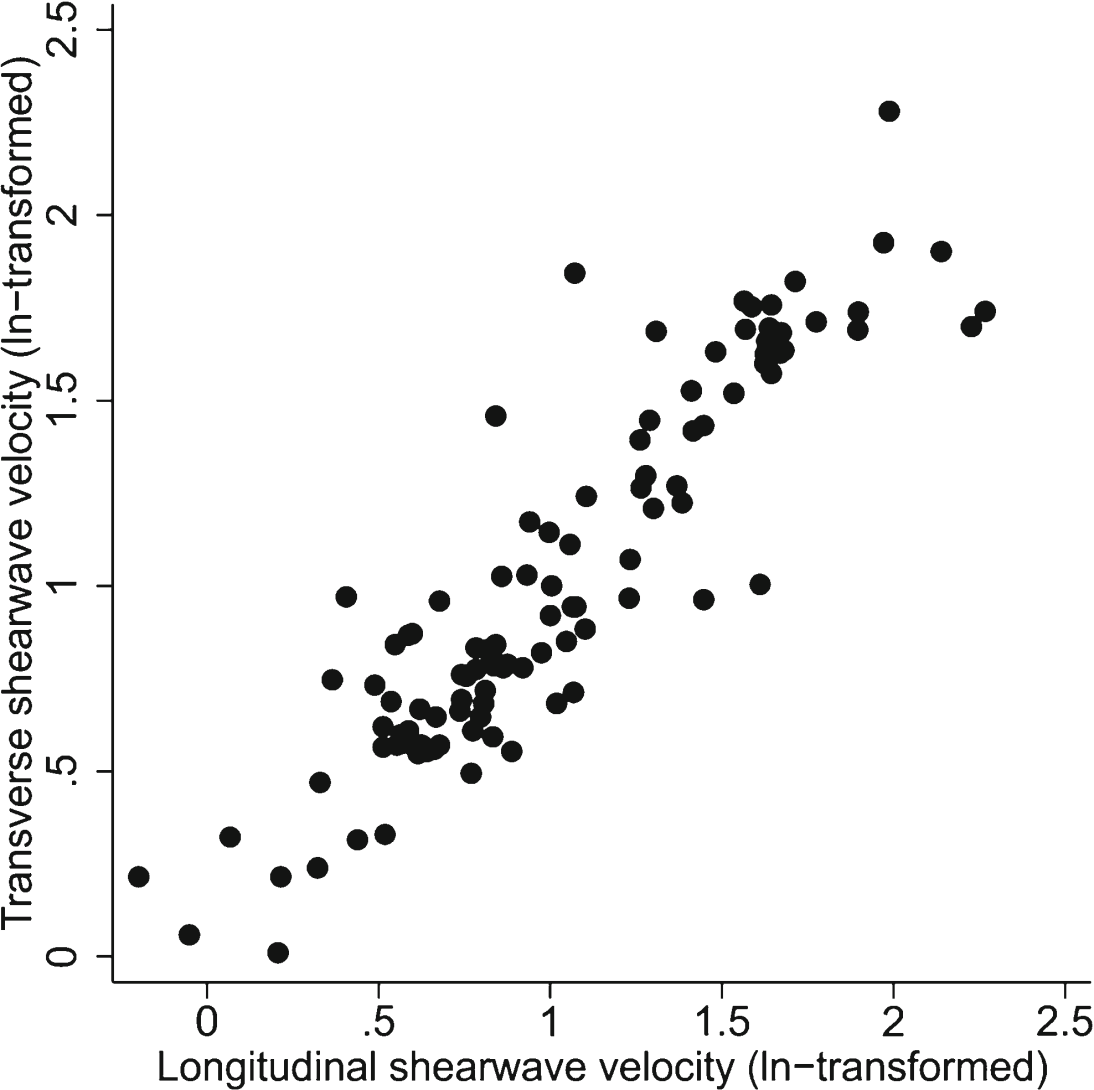
Association between shear wave velocities in the longitudinal and transverse planes (n = 105)

**Fig. 4:**
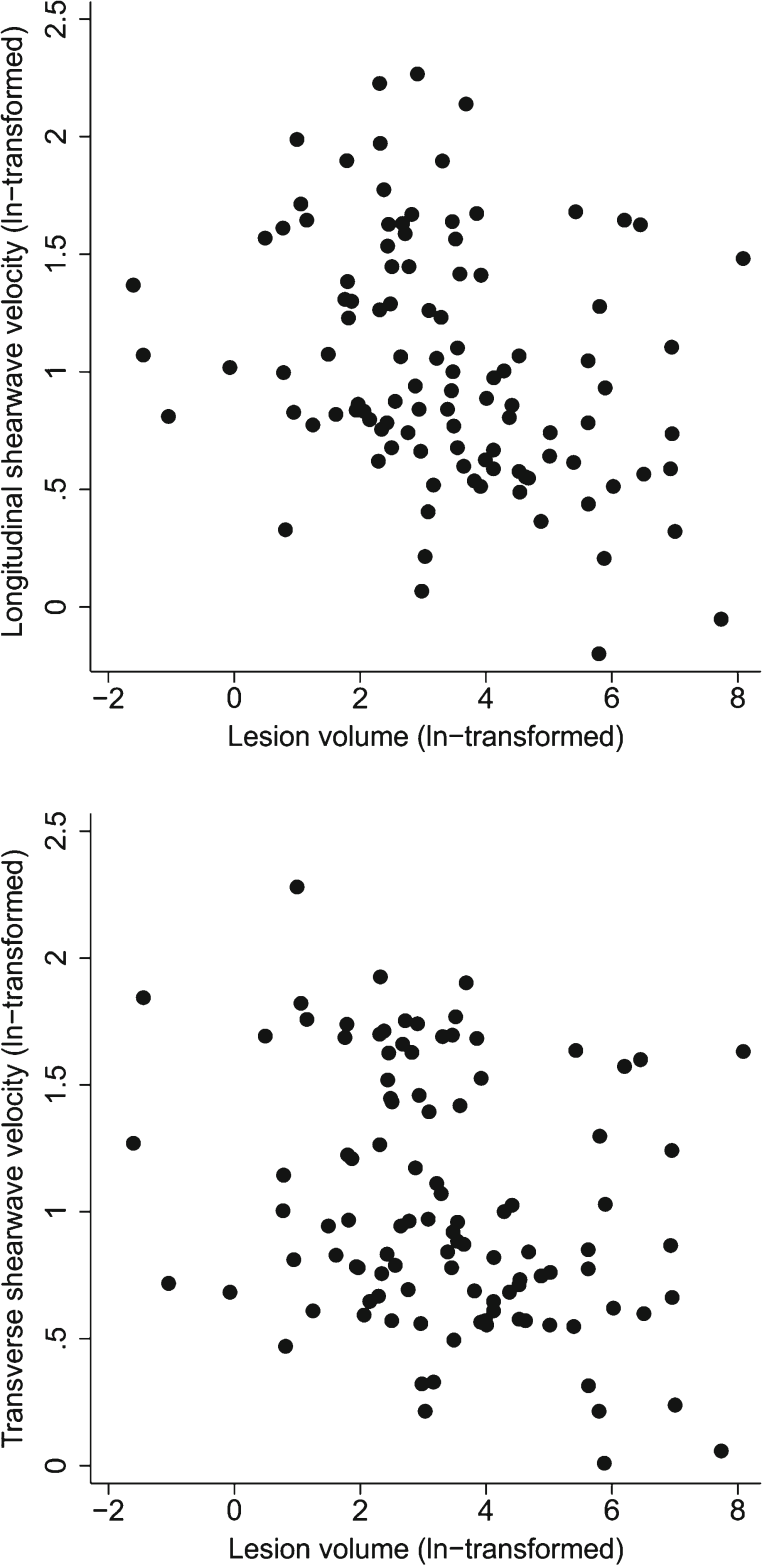
Associations between lesion volume and shear wave velocities in the longitudinal and transverse planes (n = 105)

Longitudinal and transverse shear wave velocity measurements were strongly correlated (n = 105, Pearson’s r = 0.91, p < 0.001; Fig. 3). There was a weak negative correlation between shear wave velocity and lesion volume (longitudinal r = -0.30, p = 0.001; transverse r = -0.29, p = 0.003; Fig. 4). In this unadjusted analysis, there was no evidence to suggest that velocity in either plane varied with patient age (data not shown).

Using shear wave velocities as the outcomes in multiple linear regression models, controlling for homogeneity, necrosis, echogenicity, power Doppler signal, lesion volume, and position revealed that longitudinal velocity tended to be higher in necrotic and/or heterogeneous lesions (Table 4). Having controlled for the US findings, there was around a 1 % decrease in velocity for every 10 % increase in lesion volume. Hyperechogenicity, doppler signal, and lesion position were not associated with longitudinal velocity. Results were similar in the transverse plane, but necrosis and heterogeneity were not significant even at the 10 % level.

**Table 4:** Adjusted associations between US findings and shear wave velocities in the longitudinal and transverse planes

	Ratio (95 % CI), *P* value	
	Longitudinal	Transverse
Heterogeneous vs homogenenous	1.20 (0.96, 1.50), p = 0.101	1.13 (0.89, 1.44), p = 0.318
Necrotic vs non-necrotic	1.25 (1.02, 1.54), p = 0.043	1.13 (0.92, 1.39), p = 0.238
Hyperechogenic vs hypo/mixed	0.85 (0.67, 1.07), p = 0.169	0.94 (0.70, 1.25), p = 0.659
PD linear vs PD absent	1.18 (0.93, 1.48), p = 0.169	1.20 (0.93, 1.55), p = 0.153
PD disorg vs PD absent	1.06 (0.85, 1.32), p = 0.597	1.11 (0.89, 1.40), p = 0.361
Lesion volume	-0.93 (-1.43, -0.43)*, p < 0.001	-0.82 (-1.37, -0.27)*, p = 0.004
Intramuscular vs subcutaneous	0.89 (0.71, 1.11), p = 0.293	0.92 (0.73, 1.16), p = 0.475
Intermuscular vs subcutaneous	0.89 (0.70, 1.14), p = 0.374	0.88 (0.69, 1.13), p = 0.330

*Expected % decrease in velocity per 10 % increase in volume

### Mass location and velocity

There were no substantive differences between lesions located subcutaneously, between or within muscles, in either plane (longitudinal ANOVA F_(2,102)_ = 1.24, p = 0.294; transverse F = 1.03, p = 0.360).

Expressing velocity in the two planes as a ratio (longitudinal:transverse) revealed that intramuscular masses tended to have faster longitudinal velocity relative to transverse velocity, while for lesions located elsewhere the velocities were more evenly balanced (intramuscular n = 38, median (inter-quartile range) 1.05 [0.97, 1.13]; subcutaneous n = 30, 1.00 [0.90, 1.13]; intermuscular n = 37 0.98 [0.86, 1.09]). However, overall there was not a statistically significant difference between the three groups (Kruskal-Wallis X^2^_(2)_ = 2.34, p = 0.311).

### Shear wave velocity, US findings, and diagnosis

Mean colour map pixel proportions showed a tendency for malignant masses to be towards the blue spectrum. However, the proportion of malignant lesions did not differ substantively between those that were predominantly blue (39.5 % [17/43]), cyan (46.2 % [6/13]) or yellow (42.9 % [12/28]). A smaller proportion of red lesions were malignant (14.3 % [1/7]); however, sample size was very small for this category, which means this estimate is likely to be inaccurate.

Binary logistic regression models were constructed to examine whether shear wave velocity provided additional predictive power for detection of biopsy-confirmed malignancy over and above B-mode consensus classification. Longitudinal and transverse velocities were not entered into the same model to avoid problems of multicollinearity. Instead, each was entered into a separate multivariable model (Table 5). In simple (unadjusted) analyses, older age, malignant consensus US diagnosis and greater lesion volume were all associated with significantly higher odds of confirmed malignancy. Shear wave velocities were substantively lower in malignant lesions (geometric mean m/s longitudinal benign 2.94 vs. malignant 2.57; transverse 2.93 vs. 2.56). However, the confidence intervals around the associated odds ratios were wide and crossed 1. In the first of the adjusted models (model 1) only age, US consensus classification and lesion volume were entered. In this model, a B-mode consensus classification of definitely or indeterminately malignant was significantly associated with higher odds of confirmed malignancy, with evidence (borderline-significant at 10 % level) that greater volume and older age were associated with increased odds of malignancy. Adding in longitudinal shear wave velocity (model 2) improved the model performance in terms of pseudo R-squared and AIC very slightly, but again no statistically significant association between velocity and malignancy was found, despite the odds ratio (OR) being lower in this adjusted model compared to the unadjusted OR. In model 3 there was some evidence (borderline-significant at 10 % significance level) that higher transverse velocity was associated with decreased odds of malignancy.

**Table 5:** Odds of confirmed malignancy according to US consensus classification, lesion volume, and shear wave velocity

	Biopsy confirmed diagnosis		Unadjusted OR	Adjusted OR (95 % confidence interval), *P* value		
	Benign n = 66	Malignant n = 39		Model 1: without SW velocity	Model 2: longitudinal SW velocity	Model 3: transverse SW velocity
Age						
Per year	48.1 (17.5)	59.3 (17.7)	1.04 (1.01, 1.06)	1.03 (1.00, 1.06), p = 0.091	1.03 (0.99, 1.07), p = 0.110	1.03 (0.99, 1.07), p = 0.097
US consensus						
Benign	78.8 % (52)	23.1 % (9)	Reference	Reference	Reference	Reference
Malignant	21.2 % (14)	76.9 % (30)	12.38 (4.55, 33.72)	12.38 (2.75, 55.76), p = 0.001	17.11 (2.94, 99.62), p = 0.002	19.59 (3.31, 116.01), p = 0.001
Lesion volume						
per ln(cm^3^)	2.80 (1.68)	4.35 (1.86)	1.68 (1.18, 2.40)	1.52 (0.92, 2.53), p = 0.105	1.38 (0.82, 2.30), p = 0.224	1.33 (0.79, 2.23), p = 0.285
Longitudinal SW velocity						
per ln(m/s)	1.08 (0.49)	0.95 (0.54)	0.60 (0.25, 1.40)	-	0.40 (0.10, 1.60), p = 0.193	-
Transverse SW velocity						
per ln(m/s)	1.07 (0.51)	0.94 (0.46)	0.57 (0.25, 1.29)	-	-	0.28 (0.06, 1.28), p = 0.101
				Model performance indictors		
		Pseudo R-squared		0.35	0.37	0.38
		Akaike Information Criterion		98.39	97.78	96.3
		Area under ROC curve (95 % CI)		0.87 (0.79, 0.95)	0.87 (0.80, 0.95)	0.88 (0.80, 0.95)
		Maximum YI		0.65	0.67	0.67
		Predicted probability at max YI		0.53	0.53	0.42
		Sensitivity at max YI (95 % CI)		0.74 (0.59, 0.85)	0.74 (0.59, 0.85)	0.79 (0.64, 0.89)
		Specificity at max YI (95 % CI)		0.91 (0.82, 0.96)	0.92 (0.83, 0.97)	0.88 (0.78, 0.94)
		PPV at max YI (95 % CI)		0.83 (0.67, 0.92)	0.85 (0.70, 0.94)	0.79 (0.64, 0.89)
		NPV at max YI (95 % CI)		0.86 (0.76, 0.92)	0.86 (0.76, 0.92)	0.88 (0.78, 0.94)

NPV = negative predictive value; OR = odds ratio; PPV = positive predictive value; ROC = receiver operating characteristic curve for predicted probability; SW = shear wave; YI = Youden Index ([sensitivity + specificity]-1)

Using the predicted probabilities from each model, the area under the receiver operating characteristic curve (AUC ROC) was calculated for each model. There was very little difference between the three models, indicating that adding shear wave velocity in either plane did little to improve the predictive strength of the model.

Adding shear wave velocities to models that included US consensus classification did not substantively improve diagnostic accuracy. We investigated models which included age, lesion volume, and shear wave velocity. However, in these models shear wave velocity was neither substantively nor significantly associated with malignancy (longitudinal OR [95 % CI] 0.99 [0.36, 2.71]; transverse 0.85 [0.30, 2.41]) and predictive ability was reduced (ROC [95 % CI] longitudinal 0.77 [0.67, 0.86]; transverse 0.77 [0.68, 0.87]) compared to model 1 (Table 5).

In model 1, which included age and lesion volume in addition to B-mode consensus classification but did not include shear wave velocity, AUC ROC was high at 0.87 (0.79, 0.95). Choosing a cut-point with a maximal Youden Index ([sensitivity + specificity]-1), i.e. the point at which misclassifications were minimized, yielded a sensitivity of 74 % (95 % CI 59 %, 85 %) and specificity of 91 % (82 %, 96 %). Compared to the consensus classification alone, the sensitivity was similar (cf 77 %) and the specificity was improved (cf 79 %); this difference was statistically significant (Chi-square_(2)_ = 8.20, p = 0.017). In this sample, negative predictive value was similar whether consensus scoring was used alone (85 %) or in combination with age and lesion volume (86 %) but with the addition of these variables, positive predictive value increased from 68 to 83 %.

## Discussion

Conventional imaging cannot always differentiate between malignant and benign lesions, meaning that biopsy is required to establish a diagnosis and guide management [21, 22]. The consensus B-mode US scoring demonstrated sensitivity and specificity of 76.9 and 78.8 %, confirming that it is not sufficiently accurate to be used alone in providing this differentiation, although this was under study conditions where scorers were blinded to the history and any other imaging performed. In this study, nine masses scored as benign or indeterminately benign on B-mode imaging alone were found to be malignant. Five of these were found to be grade 1 liposarcomas with normal histology but with cytogenetic analysis confirming MDM2 amplification. The limitation of radiological assessment in this particular mass is well recognised and could also potentially produce errors in elastography analysis, as the mass behaves grossly as a solid benign lesion [23]. Of the other lesions, three were also low-grade sarcomas (fibromyxoid), but one that was scored as a sebaceous cyst on B mode imaging was a high-grade pleomorphic sarcoma.

We found that in unadjusted analyses the odds of malignancy were not substantively associated with quantitative shear wave velocity with a non-significant trend for malignant masses to exhibit slower velocities. Longitudinal and transverse shear wave velocity measurements were strongly correlated. Malignancy was found to be more likely in older patients with larger lesions, necrotic lesions, and those with disorganised internal doppler vascularity.

The qualitative component of the study demonstrated that colour map readings correlated with the mean shear wave velocity, with substantial reader agreement. There was a tendency for malignant masses to be towards the blue spectrum (lower velocities), which is similar to the findings of Magarelli et al and Park et al [16, 17]. The proportion of lesions found to be malignant did not differ substantively between those lesions that where predominantly blue, cyan, or yellow, but those with predominantly red lesions were less likely to be malignant. Again, this finding is in keeping with Magarelli et al.; as in their study (n = 32), our sample size is small (n = 93), but the current study had a much higher proportion of patients with histological confirmation of diagnosis (88/93 compared to 12/32). Park et al. focused on epidermoid cysts (n = 29) showing lower strain elasticity than malignant masses (n = 20) using a subjective colour-coding system.

Elastography measurement failure rates (3.1-3.8 %) and unreliable values (7-15.8 %) have been described in the evaluation of hepatic fibrosis [24, 25]. In this current study if the lesion could be visualised on US then the shear wave velocity readings were always obtainable. The reason for this difference is unknown but may be due to the wide velocity range available (10 m/s), eliminating any aliasing error due to increased density within or adjacent to the mass.

Limitations to this study include the relatively small population size (n = 105), but this number is larger than any previous musculoskeletal series. Although it fell short of the intended sample size of 135, with 39 malignant lesions the event-per-variable ratio was still approximately 10:1 in our multivariable logistic regression models of malignancy, which contained up to four variables. Histology was available in the majority of patients (99/105), with the remaining patients having clinical and imaging follow-up. All malignant lesions had histological confirmation. Inaccuracies in the B-mode assessment may have occurred due to scoring the images in isolation without any clinical history, or previous or alternate studies (e.g. MRI).

In conclusion, this study demonstrates that whilst there may be some evidence of an association between lower shear wave velocity and soft tissue malignancy, particularly in the transverse plane, this did not result in a substantive improvement in the ability to detect malignancy over B-mode consensus classification. Qualitatively, the findings that colour maps may be more suggestive of a benign pathology if they are predominantly red and a malignancy if they are predominantly blue (lower velocities) was not discriminatory, as all colour groups contained benign and malignant masses. However, the major difference to consider when comparing elastography use in musculoskeletal soft tissue masses to thyroid and breast lesions is the much greater variation in grade and histological type that occur with sarcomas. Exact typing for sarcoma diagnosis or even its exclusion is often only made after cytogenetic or molecular analysis with differentiation not possible on imaging, or gross or histological analysis. Future studies may need to collate larger numbers of specific sarcoma sub-types and grades to evaluate this technique in this very heterogeneous tumour type.

## References

[1] Kransdorf MJ, Murphey MD. Imaging of soft tissue tumors. Philadelphia: Lippincott Williams & Wilkins; 2006. ISBN 0-7817-4771-6

[2] Clark MA, Fisher C, Judson I (2005). Soft-tissue sarcomas in adults. N Engl J Med.

[3] Clasby R, Tilling K, Smith MA (1997). Variable management of soft tissue sarcoma: regional audit with implications for specialist care. Br J Surg.

[4] Yoon JH, Kim MH, Kim EK (2011). Interobserver variability of ultrasound elastography: how it affects the diagnosis of breast lesions. Am J Roentgenol.

[5] Palmeri ML, Nightingale KR (2011). Acoustic radiation force-based elasticity imaging methods. Interface Focus.

[6] Krouskop TA, Wheeler TM, Kallel F, Garra BS, Hall T (1998). Elastic moduli of breast and prostate tissues under compression. Ultrason Imaging.

[7] Berg WA, Cosgrove DO, Doré CJ, Schäfer FK, Svensson WE, Hooley RJ (2012). Shear wave elastography improves the specificity of breast ultrasound: the BE1 Multinational Study of 939 Masses. Radiology.

[8] Ianculescu V, Ciolovan LM, Dunant A, Vielh P, Mazouni C, Delaloge S, Dromain C, Blidaru A, Balleyguier C (2014). Added value of Virtual Touch IQ shear wave elastography in the ultrasound assessment of breast lesions. Eur J Radiol.

[9] Cosgrove DO, Berg WA, Doré CJ, Skyba DM, Henry JP, Gay J, Cohen-Bacrie C, BE1 Study Group (2012). Shear wave elastography for breast masses is highly reproducible. Eur Radiol.

[10] Klauser AS, Tagliafico A, Allen GM (2012). Clinical indications for musculoskeletal ultrasound: a Delphi-based consensus paper of the European Society of Musculoskeletal Radiology. Eur Radiol.

[11] McNally EG (2011). The development and clinical applications of musculoskeletal ultrasound. Skelet Radiol.

[12] Lakkaraju A, Sinha R, Garikipati R, Edward S, Robinson P (2009). Ultrasound for initial evaluation and triage of clinically suspicious soft-tissue masses. Clin Radiol.

[13] De Zordo T, Chhem R, Smekal V (2010). Realtime sonoelastography: findings in patients with symptomatic achilles tendons and comparison to healthy volunteers. Ultraschall Med.

[14] De Zordo T, Lill SR, Fink C (2009). Real-time sonoelastography of lateral epicondylitis: comparison of findings between patients and healthy volunteers. AJR Am J Roentgenol.

[15] Silvestri E, Garlaschi G, Bartolini B (2007). Sonoelastography can help in the localization of soft tissue damage in polymyalgia rheumatica (PMR). Clin Exp Rheumatol.

[16] Magarelli N, Carducci C, Bucalo C, Filograna L, Rapisarda S, De Waure C (2014). Sonoelastography for qualitative and quantitative evaluation of superficial soft tissue lesions: a feasibility study. Eur Radiol.

[17] Park HJ, Lee SY, Lee SM, Kim WT, Lee S, Ahn KS (2015). Strain elastography features of epidermoid tumours in superficial soft tissue: differences from other benign soft-tissue tumours and malignant tumours. Br J Radiol.

[18] Benson J, Fan L. Tissue strain analytics – a complete ultrasound solution for elastography. Siemens Healthcare White Paper; 2012.

[19] Doherty JR, Trahey GE, Nightingale KR, Palmeri ML (2013). Acoustic radiation force elasticity imaging in diagnostic ultrasound. IEEE Trans Ultrason Ferroelectr Freq Control.

[20] Peduzzi P, Concato J, Kemper E, Holford TR, Feinstein AR (1996). A simulation study of the number of events per variable in logistic regression analysis. J Clin Epidemiol.

[21] Kransdorf MJ, Jelinek JS, Moser RP, Utz JA, Brower AC, Hudson TM (1989). Soft-tissue masses: diagnosis using MR imaging. AJR Am J Roentgenol.

[22] Moulton JS, Blebea JS, Dunco DM, Braley SE, Bisset GS, Emery KH (1995). MR imaging of soft-tissue masses: diagnostic efficacy and value of distinguishing between benign and malignant lesions. AJR Am J Roentgenol.

[23] Kransdorf MJ, Bancroft LW, Peterson JJ, Murphey MD, Foster WC, Temple HT (2002). Imaging of fatty tumors: distinction of lipoma and well-differentiated liposarcoma. Radiology.

[24] Roulot D, Costes JL, Buyck JF, Warzocha U, Gambier N, Czernichow S (2011). Transient elastography as a screening tool for liver fibrosis and cirrhosis in a community-based population aged over 45 years. Gut.

[25] Castéra L, Foucher J, Bernard PH, Carvalho F, Allaix D, Merrouche W (2010). Pitfalls of liver stiffness measurement: a 5-year prospective study of 13,369 examinations. Hepatology.

